# Psychosocial Distress among Family Members of COVID-19 Patients Admitted to Hospital and Isolation Facilities in the Philippines: A Prospective Cohort Study

**DOI:** 10.3390/jcm11175236

**Published:** 2022-09-05

**Authors:** Leilanie Apostol-Nicodemus, Ian Kim B. Tabios, Anna Guia O. Limpoco, Gabriele Dominique P. Domingo, Ourlad Alzeus G. Tantengco

**Affiliations:** 1 Department of Family and Community Medicine, University of the Philippines—Philippine General Hospital, Taft Avenue, Manila 1000, Philippines; 2 Institute of Biology, College of Science, University of the Philippines Diliman, Quezon City 1101, Philippines; 3 College of Medicine, University of the Philippines Manila, Manila 1000, Philippines; 4 One Hospital Command Center, Pasay 1709, Philippines

**Keywords:** anxiety, coronavirus, depression, family, mental health, pandemic

## Abstract

This study determined the psychosocial impact of COVID-19 on families of adult COVID-19 patients in isolation facilities in Metro Manila, Philippines. This prospective cohort study was conducted in COVID-19 healthcare facilities. Data collection was undertaken 2 weeks and 8 weeks after discharge. Logistic regression was performed to determine the socioeconomic and clinical factors influencing anxiety, depression, and family function. Based on HADS-P, 43.2% of the participants had anxiety symptoms, and 16.2% had depression symptoms 2 weeks after the discharge of their relative with COVID-19 infection. The prevalence of anxiety and depression significantly decreased to 24.3% and 5.4%, respectively, 8 weeks after discharge. The percentage of participants with a perceived moderate family dysfunction was 9.5% in the 2nd week and 6.8% in the 8th week post discharge. Participants with perceived severe family dysfunction increased from none to 4.1%. The most inadequate family resources for the participants were economic, medical, and educational resources. Patient anxiety (*p* = 0.010) and perceived inadequate family resources (*p* = 0.032) were associated with anxiety symptoms among family members. Patient anxiety (*p* = 0.013) and low educational attainment (*p* = 0.002) were associated with anxiety symptoms among family members 8 weeks after discharge. On the other hand, patient depression (*p* = 0.013) was a factor related to depressive symptoms among family members 2 weeks after discharge. This study provided an in-depth understanding of the mental health status of family members caring for relatives with COVID-19 infection. This can be used to guide healthcare professionals caring for COVID-19 patients and their family members.

## 1. Introduction

COVID-19 is a global pandemic caused by SARS-CoV-2. The Philippines is one of the countries in Southeast Asia that suffered greatly from COVID-19. There have been surges in cases due to the new variants and changes in the health response. Throughout the pandemic, containment and mitigation measures such as physical distancing, home quarantine, and self-isolation remain at the forefront of the country’s response [1,2]. Families have an essential role in implementing these measures to control COVID-19.

Families are the basic social unit of society [3,4]. Filipinos are known for their close-knit extended family structures. Family members are the first line of support during times of sickness [5]. During disease outbreaks, families experience emotional upheaval due to anxiety and fear of the possibility of contracting the disease [6,7,8]. This emotional distress is further exacerbated by strict infection control measures that inadvertently promote stigmatization, social isolation, and economic problems stemming from losing income due to the lack of job opportunities [5,9].

The COVID-19 pandemic has caused radical changes to the average Filipino’s life, such as loss of income and decreased social interaction due to the mitigating interventions implemented by the government [10,11]. Further psychosocial distress is likely to impact those directly affected by COVID-19 by either contracting the disease or having to take care of a family member who has been infected with COVID-19. Furthermore, COVID-19 severely affects the elderly, who require more attention and care [12,13,14].

Previous studies showed that COVID-19 infection affects the mental health of the patients [15,16]. Patients infected with COVID-19 experienced severe psychological distress, including symptoms of anxiety and depression [15,16]. Lower education level and family history of psychiatric disorder were risk factors for anxiety, and home isolation was a risk factor for depression [15]. Aside from patients, there were few studies that showed that family members taking care of COVID-19 patients also experienced mental health impacts, including anxiety and depressive symptoms [17,18,19].

COVID-19 infection negatively affected family function. Family members experienced the highest dysfunction in the areas of growth and affection [20]. Family members were dissatisfied with the support they received from their families regarding their decisions to take on new activities and directions, and the way their family members expressed affection and responded to their emotions, such as anger or love [20]. Family members, particularly parents, also reported increased family conflicts due to the pandemic [21]. However, our knowledge of the psychosocial effects of COVID-19 on the families and caregivers of COVID-19 patients in the Philippines is still limited.

Therefore, understanding the impact of COVID-19 illness on families taking care of COVID-19 patients will contribute to guidelines and policies for responding to pandemics such as COVID-19 effectively. Hence, this study determined the psychosocial impact of COVID-19 on families of adult patients in isolation facilities in Metro Manila, Philippines. We showed the proportion of COVID-19 patient family members with psychological symptoms of anxiety and depression, their perceived family function and resources, and the factors associated with psychological symptoms and family dysfunction among study participants.

## 2. Materials and Methods

### 2.1. Ethical Consideration

This study was approved by the University of the Philippines Manila Research Ethics Board (UPMREB Code: 2020-280-01) and the Philippine Department of Health Single Joint Research Ethics Board (SJREB Code: 2020-100). Written informed consent was obtained from all participants (family members of COVID-19 patients) and the patients at the beginning of the study and during the subsequent follow-up interviews.

### 2.2. Study Design

The study employed a cohort study design using quantitative methodologies. The participants were interviewed during their family member’s two-week home quarantine after discharge from the facility. A follow-up data collection was performed 8 weeks post discharge of the participants’ family member who had COVID-19 using the same procedure.

### 2.3. Sampling Design

The study employed non-probabilistic sampling. Participants were chosen as convenient from the list of patients of selected COVID-19 healthcare facilities. The selection was based on the availability of contact details and ease of coordination.

### 2.4. Study Sites

The following COVID-19 designated healthcare facilities in Metro Manila were included in the study: (1) two community isolation units (CIUs) in Metro Manila, namely the PNP Kiangan Quarantine Facility in Camp Crame, Quezon City, and the University of the Philippines Diliman Silungan Molave Quarantine Facility in Diliman, Quezon City; and (2) one hospital facility, namely the COVID-19 areas of the Philippine General Hospital (PGH) in Ermita, Manila.

### 2.5. Study Population

The study involved family members taking care of adult patients diagnosed with COVID-19 at the study sites. The inclusion criteria included: (1) age greater than or equal to 18 years; (2) family member (as previously defined) of a COVID-19 patient admitted in a hospital or quarantine facility; (3) has lived in the same household as the patient for at least 12 months before the interview; (4) involved in the care of the patient; (5) agrees to participate in the study with a signed informed consent form. The exclusion criteria included: (1) with non-consenting family members of COVID-19 patients; (2) with pre-existing clinically diagnosed psychiatric disorder before COVID-19 admission; (3) unable to provide consent due to physical or mental illness, including cognitive impairment; (4) unable to participate fully and answer questions due to physical or mental illness. Family members were able to discontinue their study participation and cancel their consent.

### 2.6. Data Collection Procedure

Data collection was undertaken through telephone or online calls with family members at 2 weeks and 8 weeks after discharge of their relative with COVID-19 infection. The research assistant or field interviewer used a semi-structured questionnaire during the data collection (interviewer-administered). Handwritten notes and voice recordings of calls using a call recording application were made. Electronic data is being stored in a well-secured data cloud for storage. If the participant warranted initial psychosocial supportive care during the interview, the research assistant or field interviewer referred the patient to the principal investigator and co-investigators. The co-investigators then provided initial counseling to the participants through telephone, online voice, or video calls. If further management was necessary, the participant was referred to the Family Health Unit (FHU) clinic at the Outpatient Department of the Philippine General Hospital.

### 2.7. Data Collection Tools

In this study, quantitative data were obtained to determine the impact of the COVID-19 experience on families caring for COVID-19 patients. The questionnaire was pre-tested practically with 20 family members of COVID-19 patients who were not included in this study, to increase data quality before data collection began. The assessment tools used in the study have validated Filipino translation.

#### 2.7.1. Hospital Anxiety and Depression Scale (HADS)

Psychological symptoms of anxiety and depression were assessed using the Hospital Anxiety and Depression Scale (HADS). It is a self-report instrument designed to detect symptoms related to anxiety and depression. Initially designed for hospitalized patients, HADS has been used and validated in community settings and primary care practice. In the Philippines, a Filipino translation (HADS-P) has been validated among Filipino patients. It has fourteen items and two subscales, anxiety and depression, and each item is scored on a four-point scale of 0 to 3. A score of ≥11 was interpreted as positive for the emotional illness being tested since similar studies which used HADS/HADS-P utilized the same cut-off [22,23,24]. This tool has been used and validated in previous studies using telephone interviews with a Cronbach alpha of 0.76 to 0.86 [25].

#### 2.7.2. Family Assessment Tools

The following family assessment tools were used in the study: Filipino family APGAR (Adaptability, Partnership, Growth, Affection, and Resolve), and SCREEM (Social, Cultural, Religious, Economic, Education, and Medical) Family Resources Survey (SCREEM-RES). Filipino family APGAR, a translated and validated Filipino version of Smilkstein’s family APGAR, was used to assess family functioning based on five parameters: Adaptability, Partnership, Growth, Affection, and Resolve. Each parameter is scored with a three-point scale ranging from 0 (hardly ever) to 2 (almost always). The total scores range from 0 to 10, with higher scores indicating higher satisfaction with family functioning. A score of 0–3 shows severe family dysfunction, 4–7 moderate family dysfunction, and 8–10 highly functional families [26].

The SCREEM Family Resources Survey (SCREEM-RES) is a validated and reliable tool to measure family resources used to cope with difficult situations. This instrument is a brief twelve-item questionnaire containing all the six original SCREEM domains and includes two items per domain. Participants were asked to choose one of the following responses: strongly disagree, disagree, agree, and strongly agree. The total SCREEM-RES scores were grouped using the following key: Severely Inadequate Family Resources = 0 to 12, Moderately Inadequate Family Resources = 13 to 24, Adequate Family Resources = 25 to 36. For each domain subscale, scores were grouped using the following key: Severely Inadequate Family Resources = 0 to 2, Moderately Inadequate Family Resources = 3 to 4, Adequate Family Resources = 5 to 6. This was previously validated in Filipino patients with a Cronbach’s alpha of 0.80 for the entire scale [26].

### 2.8. Data Analysis

Data were analyzed using SPSS statistical software version 28.0 (IBM Corp). Statistical significance was set at α = 0.05, and all tests were two-tailed. Collected data were summarized using descriptive statistics, tables, and graphs. Means, medians, standard deviations, and interquartile ranges were computed for continuous variables, whereas frequencies and percentages were obtained from categorical variables. The internal consistencies of the questionnaires were reported as Cronbach alpha coefficients. Statistical comparisons between continuous variables were performed with an independent Student t-test for normally distributed data, whereas a Mann–Whitney U test was used if otherwise. A χ^2^ test or Fisher’s exact test was done for categorical variables. To check for statistical differences in the proportions of mental and social outcomes between 2 and 8 weeks after discharge, a paired-samples proportion test using McNemar was used. Binary logistic regression analysis was performed to determine factors influencing anxiety, depression, and family dysfunction symptoms. Associations between the exposure and outcome variables are presented as odds ratios and 95% CIs, after adjustment for confounders defined as exposure variables with *p* > 0.25 based on univariate analysis.

## 3. Results

### 3.1. Sociodemographic Characteristics of the Participants

A total of 104 participants were recruited for the project and completed the first interview, and 74 participants completed the second interview. Baseline sociodemographic characteristics of the 74 participants who completed the first and second interviews are shown in Table 1.

### 3.2. Dynamics of Anxiety and Depression among Family Members of COVID-19 Patients

At the cut-off HADS-P anxiety score of 11, 43.2% of the participants had anxiety symptoms 2 weeks after discharge of their relative with COVID-19 infection. The prevalence of anxiety significantly decreased to 24.3% 8 weeks after discharge (*p* = 0.002). At the cut-off HADS-P depression score of 11, 16.2% of the participants had symptoms of depression 2 weeks after the discharge of their relatives with COVID-19 infection. The prevalence of depression significantly decreased to only 5.4% at 8 weeks post discharge (*p* = 0.021). Lastly, 13.5% of the participants had a mixed diagnosis of anxiety and depression at 2 weeks and 4.1% at 8 weeks post discharge (*p* < 0.001) (Table 2 and Table S1).

Among participants with anxiety symptoms at 2 weeks post discharge, 53.1% had resolved symptoms, and 46.9% had persistent anxiety symptoms at the 8th-week post-discharge follow-up. Most participants without anxiety symptoms at the second-week follow-up remained asymptomatic during the 8th-week follow-up. However, 7.1% developed new-onset anxiety symptoms during the 8th-week follow-up (Table 3). Among participants with depression 2 weeks post discharge, 83.3% had resolved symptoms, and only 16.7% had persistent symptoms of depression during the 8th-week follow-up. Most of the participants (96.8%) without symptoms of depression during the 2nd-week follow-up remained asymptomatic, whereas 3.2% developed new-onset symptoms of depression during the 8th-week follow-up (Table 3).

### 3.3. Perceived Family Dysfunction

The Family APGAR index was used to measure the general family function. The percentage of participants with a perceived moderate family dysfunction decreased from 9.5% at 2 weeks post discharge to 6.8% at 8 weeks post discharge, whereas those with perceived severe dysfunction increased from none to 4.1% from the 2nd week to 8th week post discharge (Table 4 and Table S1). However, these observed changes did not reach statistical significance.

Analysis of the dynamics of perceived family dysfunction showed that among those with perceived dysfunction at 2 weeks, 57.1% retained the same view at 8 weeks after discharge. On the other hand, 7.5% of those without perceived dysfunction at 2 weeks developed perceived family dysfunction 8 weeks post discharge (Table 5).

### 3.4. Perceived Inadequacy of Family Resources

Among the resources measured by the SCREEM-RES questionnaire, the most inadequate resources for the family member participants were the economic, medical, and educational resources. The prevalence of perceived economic resource inadequacy decreased at 8 weeks post discharge. However, medical inadequacy increased. The resources least perceived to be inadequate were social, cultural, and religion. Perceived inadequacy in these resources increased at 8 weeks compared with 2 weeks after discharge. There was no increase in the overall perceived inadequacy from 2 weeks to 8 weeks post discharge (Table 6 and Table S1).

Analysis of the dynamics of perceived inadequacy of family resources showed that most participants with perceived inadequacy at 2 weeks retained the same view 8 weeks after discharge (59.3%). Meanwhile, 25.5% of those without perceived inadequacy at 2 weeks developed it 8 weeks post discharge (Table 7).

### 3.5. Factors Influencing Psychological Impact of COVID-19 Experience

At 2 weeks after discharge, patient anxiety (*p* = 0.010) and perceived inadequate family resources (*p* = 0.032) were associated with anxiety symptoms among family members. Patient anxiety (*p* = 0.013) and low educational attainment (*p* = 0.002) were associated with anxiety symptoms among family members 8 weeks after discharge. On the other hand, patient depression (*p* = 0.013) was a factor related to depressive symptoms among family members 2 weeks after discharge (Table 8). Using multivariate logistic regression analysis, no identified factors were associated with depressive symptoms among family members at 8 weeks after discharge.

## 4. Discussion

This prospective cohort study revealed a high prevalence of psychosocial symptoms among participants at 2 and 8 weeks after the discharge of their family member admitted for COVID-19 infection. Overall, family function and family resources contributed to anxiety and depression among patients and families post COVID. The study explored the impact of COVID-19 on the psychological symptoms of anxiety and depression and its associated sociodemographic, economic, and clinical factors. Anxiety was high in family members at 2 weeks and 8 weeks post COVID infection. Similarly, common mental health conditions such as anxiety, depression, post-traumatic stress disorder, and overall lower quality of life occur for up to 3 months or 12 weeks post COVID-19 infection.

The unpredictability of COVID-19 infection contributes to anxiety and depression among family members of patients with COVID-19. It impairs work, family engagements, and health [27]. Families may experience high stress, anxiety, and financial burden from missing work and unemployment concerns [28]. Moreover, family members’ anxiety also stemmed from their inability to feel connected to the patient and informed about care [29]. During the pandemic, family members were not allowed to stay and visit their relatives who were admitted to the hospital for COVID-19. Family members struggled to feel informed about the care they could not witness and had difficulty understanding information. A previous study showed that visits to COVID-19 patients in the ICU reduced anxiety among family members [19]. Another study showed that the family members of COVID-19 patients experienced mental health symptoms 12 months after ICU admission of their relatives for COVID-19 infection. Family members also experienced disruption of quality of life and work-related problems [17].

In this study, risk factors identified for anxiety among family members were patient depression and a low level of educational attainment. Similar findings have been made in other studies on family members of COVID-19 patients [18,29]. COVID-19 has resulted in massive unemployment worldwide. Several studies have already shown that the unemployment rate increased negative mental health outcomes during the COVID-19 pandemic [30,31,32]. It exacerbated pre-existing mental health disorders and created new disorders for others [32]. Collectively, these data showed that the government and the health care system should support patients’ families financially.

The family function measurements taken using APGAR scores showed that the participants’ perceived family dysfunction increased during the period from 2 weeks to 8 weeks after the discharge of their family member. This suggests that family dysfunction exacerbated by admission of a family member to a health facility due to COVID-19 could have long-term effects. Previous studies showed that social and family relationships were disrupted for patients and their caregivers [33]. The stresses created by the COVID-19 pandemic have put families and their interrelationships under tremendous pressure [34]. A previous study in Portugal showed that almost 20% of the participants perceived their families to have severe dysfunction or moderate dysfunction [20]. Family dysfunction is a predisposing factor for developing the emotional problems of anxiety and depression during the COVID-19 pandemic [35].

Interestingly, despite the decrease in the prevalence of anxiety and depression in the participants during the period from 2 weeks to 8 weeks after discharge, perceived severe family dysfunction still increased. We surmised that family-level dysfunction manifests later than personal-level conditions, such as anxiety and depression. The family members and the patient need to adapt to the consequences of COVID-19 infection [36], such as long COVID-19 symptoms, financial responsibility accrued from hospitalization, loss of productivity due to inability to go to work, and unemployment. Caregiver fatigue may occur later, hence, the appearance of severe family dysfunction later after the patient’s discharge. Previous studies showed that the COVID-19 pandemic changed the structure and routine of the family, especially for those who suffered from the disease [21,37,38]. The disruptions in the usual routine resulted in physical and mental health problems and family matters [37]. Family members, particularly parents, also reported increased family conflicts due to the pandemic [21].

The perceived inadequate family resources did not decrease 8 weeks post COVID-19 discharge. The economic burden did not decrease during the period from 2 weeks to 8 weeks post discharge. Financial factors may contribute to severe family dysfunction, as shown by the APGAR score. COVID-19 has generated a considerable economic and financial burden on patients. Aside from the high cost of hospitalization, long-term health effects of COVID-19 such as kidney disease and long COVID-19 symptoms may induce chronic medical needs that expose patients and their families to long-term financial risk [39,40]. A previous study conducted in the Philippine General Hospital showed that the average out-of-pocket payment for COVID-19 patients less than 60 years old ranged from Php 25,899 to Php 44,428.63 ($538 to $924.44), whereas for patients older than 60 this ranged from Php 4005.60 to Php 32,920.20 ($83.35 to $684.98) [41]. Despite the financial help from national health insurance in the Philippines, the patients still need to pay out of pocket. This puts a financial burden on the patients and their families, especially since the daily minimum wage of an average worker in the National Capital Region of the Philippines only ranges from Php 533 to Php 570 ($9.54 to $10.20) [42].

The participants perceived a significant lack of access to medical resources at 8 weeks post discharge. The persistent perception of the participants of having inadequate access to medical resources is not surprising. The pandemic has brought disruption and barriers to accessing medical care. The availability of healthcare services related to COVID-19 disease and other chronic diseases has deteriorated due to the diversion of health services for urgent COVID-19 cases [43,44]. This lack of access to medical care was more pronounced among those belonging to the lower socioeconomic strata [45,46].

This study also showed that inadequate family resources and low educational attainment were associated with anxiety in the participants. Previous studies showed that low educational levels were significantly associated with both anxiety and depression [47,48]. On the other hand, higher educational attainment is protective against developing a spectrum of psychiatric disorders [48]. Higher educational attainment is also associated with higher income [49] and being more capable of shouldering medical expenses from COVID-19 hospitalization. The material advantage is protective of the negative effect of COVID-19 on the mental health of individuals, as shown in a previous study [50].

This study supports the need for more holistic COVID-19 practice guidelines to include psychosocial interventions among family caregivers. We emphasize the need to have family assessments in routine medical history taking. The use of validated tools for early detection and screening of mental disorders such as the Patient Health Questionnaire (PHQ) and Depression, Anxiety, and Stress Scale (DASS) is recommended. In addition, the Family APGAR and SCREEM-RES are good family assessment tools to check functional family relationships and family resource adequacy. Healthcare professionals should involve family members during active treatment and post-COVID-19 care of hospitalized patients and those in the quarantine facilities as part of treatment protocols. A multi-disciplinary approach to the active and follow-up care of COVID-19 patients and their family caregiver is needed. The care team must include health professionals who can provide psychological, social support, and home care.

This study has several limitations. The study only recruited family members of patients from selected COVID-19 healthcare facilities within Metro Manila, the epicenter of the pandemic in the Philippines. Experiences in areas outside Metro Manila and other metropolitan cities, where healthcare facilities have significantly different situations, may vary substantially from those recorded in the study. Second, the structural distance inherent in telephone interviews affected the engagement and retention of samples of the study because of the absence of an interpersonal relationship commonly established in face-to-face interviews. Lastly, symptoms of anxiety and depression were detected using a screening tool, and the presence of either anxiety or depression disorders was not confirmed with a diagnostic tool commonly used in psychiatry, such as DSM-5.

## 5. Conclusions

This study provided a valuable in-depth understanding of the mental health status of family members caring for relatives with COVID-19 infection. This study described the prevalence of and factors associated with psychological distress, particularly symptoms of anxiety and depression, in family members of patients with COVID-19 admitted to hospital and quarantine facilities. They experienced symptoms of anxiety and depression even after the discharge of their relative with COVID-19 infection. They also perceived moderate to severe family dysfunction and inadequacy of economic, medical, and educational resources in the family. These symptoms and perceptions persisted for 2 to 8 weeks after the discharge of their relative with COVID-19 infection. Depressive symptoms in a relative with COVID-19 infection tend to influence the occurrence of anxiety among family members. Our findings can be used to guide healthcare professionals caring for COVID-19 patients and their family members. COVID-19 infection generates a secondary public health crisis through stress-related disorders among family members of COVID-19 patients. Therefore, relevant interventions are recommended.

## Figures and Tables

**Table 1:** Baseline sociodemographic characteristics of the family members of patients who completed the two interviews (*n* = 74).

Characteristic	Participants
Age in years, mean (SD)	41.2 (11.8)
Age group, *n* (%)	
18 to 34 years old	23 (31.1)
35 to 49 years old	30 (40.5)
50 to 64 years old	19 (25.7)
65 years old and above	2 (2.7)
Sex assigned at birth, *n* (%)	
Female	49 (66.2)
Male	25 (33.8)
Health care facility, *n* (%)	
PGH COVID-19 Designated Referral Center	42 (56.8)
UP Diliman Silungan Molave Quarantine Facility	22 (29.7)
PNP Kiangan Quarantine Facility	10 (13.5)
COVID-19 severity of relative during admission, *n* (%)	
Critical and severe	24 (32.4)
Moderate	11 (14.9)
Mild	35 (47.3)
Asymptomatic	4 (5.4)
Civil status, *n* (%)	
Married	45 (60.8)
Cohabitation	9 (12.2)
Separated	1 (1.4)
Widow	1 (1.4)
Single	18 (24.3)
Relationship with the patient, *n* (%)	
Romantic partner	35 (47.3)
Parent	16 (21.6)
Sibling	17 (23.0)
Close relatives (cousin, niece, nephew, aunt, uncle, etc.)	6 (8.1)
Educational attainment, *n* (%)	
Post-graduate	2 (2.7)
College	48 (64.9)
Vocational	7 (9.5)
Secondary school	15 (20.3)
Primary school	2 (2.7)
Employment status, *n* (%)	
Regular, *n* (%)	30 (40.5)
Self-employed, *n* (%)	7 (9.5)
Contractual, *n* (%)	15 (20.3)
Unemployed, *n* (%)	22 (29.7)
Number of household members, median (IQR)	5 (4–6)
Number of household members, *n* (%)	
less than 5	32 (43.2)
5 or more	42 (56.8)
Diagnosed with at least one chronic disease, *n* (%)	33 (44.6)
Had previous hospital admission, *n* (%)	36 (48.6)
Had previous surgery, *n* (%)	33 (44.6)
Income classification based on PIDS 2018, *n* (%)	
Poor (monthly salary below ₱ 10,957.0)	15 (20.3)
Low income (monthly salary of ₱ 10,957.0 to 43,828.0)	31 (41.9)
Middle income (monthly salary of ₱ 43,828 to 219,140)	28 (37.8)
Knew someone who died due to COVID-19, *n* (%)	21 (28.4)
Knew someone else who had COVID-19, *n* (%)	42 (56.8)

Abbreviations: SD, standard deviation; IQR, interquartile range; PGH, Philippine General Hospital; PNP, Philippine National Police; UP, University of the Philippines; PIDS, Philippine Institute for Development Studies; ND, No Data.

**Table 2:** The proportion of family members with symptoms of anxiety and depression at 2 and 8 weeks after discharge of their relatives with COVID-19 from the study sites (*n* = 74).

Mental Health Outcomes	2 Weeks after Discharge		8 Weeks after Discharge		*p* Value
	*n*	% (95% CI)	*n*	% (95% CI)	
Anxiety	32	43.2 (31.7–55.3)	18	24.3 (15.7–35.0)	0.002
Depression	12	16.2 (8.7–26.6)	4	5.4 (1.9–12.3)	0.021
Mixed diagnosis, *n* (%)	10	13.5 (6.7–23.5)	3	4.1 (1.2–10.4)	<0.001

**Table 3:** Dynamics of anxiety and depressive symptoms among family members at 2 and 8 weeks after discharge (*n* = 74).

Psychosocial Condition	Symptomatic at 2 Weeks			Asymptomatic at 2 Weeks		
	*n*	Resolved Symptoms at 8 Weeks *n* (%)	Remained Symptomatic at 8 Weeks *n* (%)	*n*	Remained Asymptomatic at 8 Weeks *n* (%)	Developed Symptoms at 8 Weeks *n* (%)
Anxiety	32	17 (53.1)	15 (46.9)	42	39 (92.9)	3 (7.1)
Depression	12	10 (83.3)	2 (16.7)	62	60 (96.8)	2 (3.2)

**Table 4:** The proportion of family members with perceived family dysfunction at 2 and 8 weeks after discharge of their relatives with COVID-19 from the study sites (*n* = 74).

Social Outcome	2 Weeks after Discharge		8 Weeks after Discharge		*p* Value
	*n*	% (95% CI)	*n*	% (95% CI)	
Dysfunctional	7	9.5 (3.8–18.5)	8	10.8 (4.8–20.2)	0.655
Moderately	7	9.5 (3.8–18.5)	5	6.8 (2.6–14.2)	
Severely	0	0 (0.1–4.4)	3	4.1 (1.2–10.4)	

**Table 5:** Dynamics of perceived family dysfunction among adult patients and family members at 2 and 8 weeks after discharge of their relatives with COVID-19 from the study sites (*n* = 74).

Psychosocial Condition	With Dysfunction at 2 Weeks			Without Dysfunction at 2 Weeks		
	*n* (%)	Resolved at 8 Weeks *n* (%)	Remained with Dysfunction at 8 Weeks *n* (%)	*n* (%)	Remained at 8 Weeks *n* (%)	Developed Dysfunction at 8 Weeks *n* (%)
Family dysfunction	7 (9.5)	3 (4.1)	4 (5.4)	67 (90.5)	62 (83.78)	5 (6.8)

**Table 6:** The proportion of patients’ family members with perceived inadequate family resources at 2 and 8 weeks after discharge of their relatives with COVID-19 infection from the study sites (*n* = 74).

Social Outcome	2 Weeks after Discharge		8 Weeks after Discharge		*p* Value
	*n*	% (95% CI)	*n*	% (95% CI)	
**Overall Resources**					
Inadequate	25	33.8 (23.2–45.7)	28	36.5 (25.6–48.5)	0.827
Moderate	25	33.8 (23.2–45.7)	26	35.1 (25.0–46.4)	
Severe	0	0 (0.0–2.4)	2	2.7 (0.6–8.4)	
**Social Resources**					
Inadequate	27	36.5 (25.6–48.5)	27	36.5 (25.6–48.5)	0.819
Moderate	27	36.5 (25.6–48.5)	26	35.1 (25.0–46.4)	
Severe	0	0 (0.0–2.4)	1	1.4 (1.0–6.1)	
**Cultural Resources**					
Inadequate	31	42.3 (33.1–51.9)	38	51.4 (39.4–63.2)	0.072
Moderate	31	42.3 (33.1–51.9)	33	44.6 (33.7–55.9)	
Severe	0	0 (0.0–2.4)	5	6.8 (2.6–14.2)	
**Religion Resources**					
Inadequate	30	40.5 (29.3–52.6)	28	37.8 (26.8–49.9)	0.467
Moderate	28	37.8 (26.8–49.9)	26	35.1 (25.0–46.4)	
Severe	2	2.7 (0.3–9.4)	2	2.7(0.6–8.4)	
**Economic Resources**					
Inadequate	57	77.0 (65.8–86.0)	56	75.7 (64.3–84.9)	0.617
Moderate	45	60.8 (48.7–72.0)	44	59.5 (48.1–70.1)	
Severe	12	16.2 (8.7–26.6)	12	16.2 (9.2–25.8)	
**Educational Resources**					
Inadequate	42	56.8 (44.7–68.2)	46	62.2 (50.1–72.2)	0.532
Moderate	38	51.4 (39.4–63.2)	42	56.8 (45.4–67.6)	
Severe	4	5.4 (1.5–13.3)	4	5.4 (1.9–12.3)	
**Medical Resources**					
Inadequate	61	82.4 (71.8–90.3)	57	77.0 (65.8–86.0)	0.225
Moderate	44	59.5 (47.4–70.7)	39	52.7 (41.4–63.8)	
Severe	17	23.0 (14.0–34.2)	18	24.3 (15.7–35.0)	

**Table 7:** Dynamics of perceived inadequacy of family resources among patients and family members at 2 and 8 weeks after discharge (*n* = 74).

Psychosocial Condition	With Perceived Inadequate Family Resources at 2 Weeks			Without Perceived Inadequate Family Resources at 2 Weeks		
	*n*	Resolved at 8 Weeks *n* (%)	Remained with Inadequacy at 8 Weeks *n* (%)	*n*	Remained at 8 Weeks *n* (%)	Developed Perceived Inadequacy at 8 Weeks *n* (%)
Inadequate family resources	27	11 (14.9)	16 (21.6)	47	35 (47.3)	12 (16.2)

**Table 8:** Factors associated with psychological symptoms in patients’ family members identified by multivariate logistic regression analysis.

Explanatory Variable	Adjusted Odds Ratio (95% CI)	*p* Value
**Condition: Anxiety in Family Members at 2 Weeks after Facility Discharge** ^1^		
Patient anxiety at 8 weeks after facility discharge		
With anxiety	34.3 (2.3–500.5)	0.010
No anxiety	1	
Family resources at 8 weeks after facility discharge		
Inadequate	6.5 (1.2–35.7)	0.032
Adequate	1	
**Condition: Anxiety in Family Members at 8 Weeks after Facility Discharge** ^2^		
Patient anxiety at 2 weeks after facility discharge		
With anxiety	5.9 (1.5–23.8)	0.013
No anxiety	1	
Highest educational attainment		
Lower than high school	0.1 (0.0–0.4)	0.002
College or higher	1	
**Condition: Depression in Family Members at 2 Weeks after Facility Discharge** ^3^		
Patient depression at 2 weeks after facility discharge		
With depression	18.0 (1.8–176.4)	0.013
No depression	1	

^1^ Adjusted for number of household members, diagnosed with chronic disease, employment status, perceived family functioning at 2 and 8 weeks after facility discharge, social resources at 2 weeks after facility discharge, overall family resources at 8 weeks after facility discharge, presence of patient anxiety at 2 and 8 weeks after facility discharge; ^2^ adjusted for highest educational attainment, perceived family functioning at 2 weeks after facility discharge, patient anxiety and depression at 2 weeks after facility discharge; ^3^ adjusted for marital status, number of household members, presence of chronic disease, knew somebody who died due to COVID-19, perceived family functioning at 2 weeks after facility discharge, medical resources at 2 weeks after facility discharge, patient anxiety and depression at 2 and 8 weeks after facility discharge.

## Data Availability

The data that supports the findings of this study are available in the Supplementary Material of this article.

## Supplementary Materials

The following supporting information can be downloaded at: https://www.mdpi.com/article/10.3390/jcm11175236/s1, Table S1: Instrument scores of patients’ family members at 2 and 8 weeks after discharge of their relatives with COVID-19 from the study sites (*n* = 74).

## References

[1] Amit A.M.L., Pepito V.C.F., Dayrit M.M. (2021). Early Response to COVID-19 in the Philippines. West. Pacific Surveill. Response J. WPSAR.

[2] S Talabis D.A., Babierra A.L.H., Buhat C.A., Lutero D.S., Quindala K.M., Rabajante J.F. (2021). Local Government Responses for COVID-19 Management in the Philippines. BMC Public Health.

[3] Akhmedov B.T. (2021). The Family as the Basic Unit of Society. Int. J. Multicult. Multirelig. Underst..

[4] Ebrahim G.J. (1982). The Family as a Child-Rearing Unit of Society. Child Health in a Changing Environment.

[5] Rahimi T., Dastyar N., Rafati F. (2021). Experiences of Family Caregivers of Patients with COVID-19. BMC Fam. Pract..

[6] Wister A., Li L., Mitchell B., Wolfson C., McMillan J., Griffith L.E., Kirkland S., Raina P. (2022). Levels of Depression and Anxiety Among Informal Caregivers during the COVID-19 Pandemic: A Study Based on the Canadian Longitudinal Study on Aging. J. Gerontol. Ser. B.

[7] Irani E., Niyomyart A., Hickman R.L. (2021). Family Caregivers’ Experiences and Changes in Caregiving Tasks during the COVID-19 Pandemic. Clin. Nurs. Res..

[8] Cohen S.A., Kunicki Z.J., Drohan M.M., Greaney M.L. (2021). Exploring Changes in Caregiver Burden and Caregiving Intensity Due to COVID-19. Gerontol. Geriatr. Med..

[9] Wittenberg E., Saada A., Prosser L.A. (2013). How Illness Affects Family Members: A Qualitative Interview Survey. Patient.

[10] Tee M.L., Tee C.A., Anlacan J.P., Aligam K.J.G., Reyes P.W.C., Kuruchittham V., Ho R.C. (2020). Psychological Impact of COVID-19 Pandemic in the Philippines. J. Affect. Disord..

[11] Aruta J.J.B.R., Callueng C., Antazo B.G., Ballada C.J.A. (2022). The Mediating Role of Psychological Distress on the Link between Socio-Ecological Factors and Quality of Life of Filipino Adults during COVID-19 Crisis. J. Community Psychol..

[12] Agrupis K.A., Smith C., Suzuki S., Villanueva A.M., Ariyoshi K., Solante R., Telan E.F., Estrada K.A., Uichanco A.C., Sagurit J. (2021). Epidemiological and Clinical Characteristics of the First 500 Confirmed COVID-19 Inpatients in a Tertiary Infectious Disease Referral Hospital in Manila, Philippines. Trop. Med. Health.

[13] Malundo A.F.G., Abad C.L.R., Salamat M.S.S., Sandejas J.C.M., Planta J.E.G., Poblete J.B., Morales S.J.L., Gabunada R.R.W., Evasan A.L.M., Cañal J.P.A. (2022). Clinical Characteristics of Patients with Asymptomatic and Symptomatic COVID-19 Admitted to a Tertiary Referral Centre in the Philippines. IJID Reg..

[14] Salva E.P., Villarama J.B., Lopez E.B., Sayo A.R., Villanueva A.M.G., Edwards T., Han S.M., Suzuki S., Seposo X., Ariyoshi K. (2020). Epidemiological and Clinical Characteristics of Patients with Suspected COVID-19 Admitted in Metro Manila, Philippines. Trop. Med. Health.

[15] Mohamed A.E., Yousef A.M. (2021). Depressive, Anxiety, and Post-Traumatic Stress Symptoms Affecting Hospitalized and Home-Isolated COVID-19 Patients: A Comparative Cross-Sectional Study. Middle East Curr. Psychiatry.

[16] Moayed M.S., Vahedian-Azimi A., Mirmomeni G., Rahimi-Bashar F., Goharimoghadam K., Pourhoseingholi M.A., Abbasi-Farajzadeh M., Hekmat M., Sathyapalan T., Guest P.C. (2021). Depression, Anxiety, and Stress Among Patients with COVID-19: A Cross-Sectional Study. Adv. Exp. Med. Biol..

[17] Heesakkers H., van der Hoeven J.G., Corsten S., Janssen I., Ewalds E., Burgers-Bonthuis D., Rettig T.C.D., Jacobs C., van Santen S., Slooter A.J.C. (2022). Mental Health Symptoms in Family Members of COVID-19 ICU Survivors 3 and 12 Months after ICU Admission: A Multicentre Prospective Cohort Study. Intensive Care Med..

[18] Khaleghparast S., Ghanbari B., Maleki M., Zamani F., Peighambari M.-M., Karbalaie Niya M.H., Mazloomzadeh S., Safarnezhad Tameshkel F., Manshouri S. (2022). Anxiety, Knowledge and Lived Experiences of Families with COVID-19 Patients: A Mixed-Method Multi-Center Study in Iran. Iran. J. Med. Sci..

[19] Bartoli D., Trotta F., Pucciarelli G., Simeone S., Miccolis R., Cappitella C., Rotoli D., Rocco M. (2022). The Lived Experiences of Family Members Who Visit Their Relatives in COVID-19 Intensive Care Unit for the First Time: A Phenomenological Study. Heart Lung.

[20] Fernandes C.S., Magalhães B., Silva S., Edra B. (2020). Perception of Family Functionality during Social Confinement by Coronavirus Disease 2019. J. Nurs. Health.

[21] Gadermann A.C., Thomson K.C., Richardson C.G., Gagné M., McAuliffe C., Hirani S., Jenkins E. (2021). Examining the Impacts of the COVID-19 Pandemic on Family Mental Health in Canada: Findings from a National Cross-Sectional Study. BMJ Open.

[22] Snaith R.P. (2003). The Hospital Anxiety And Depression Scale. Health Qual. Life Outcomes.

[23] Tan S.M., Benedicto J., Santiaguel J.M. (2015). Prevalence of Anxiety and Depression among Filipino Patients with Chronic Obstructive Pulmonary Disease: A Multi-Center Study. Philipp. J. Intern. Med..

[24] De Guzman M.L.R.E. (2013). A Validation of the Hospital Anxiety and Depression Scale (HADS) in the Medically-Ill. Acta. Med. Philipp..

[25] Hedman E., Ljótsson B., Blom K., El Alaoui S., Kraepelien M., Rück C., Andersson G., Svanborg C., Lindefors N., Kaldo V. (2013). Telephone versus Internet Administration of Self-Report Measures of Social Anxiety, Depressive Symptoms, and Insomnia: Psychometric Evaluation of a Method to Reduce the Impact of Missing Data. J. Med. Internet Res..

[26] Panganiban-Corales A.T., Medina M.F.J. (2011). Family Resources Study: Part 1: Family Resources, Family Function and Caregiver Strain in Childhood Cancer. Asia Pac. Fam. Med..

[27] Trougakos J.P., Chawla N., McCarthy J.M. (2020). Working in a Pandemic: Exploring the Impact of COVID-19 Health Anxiety on Work, Family, and Health Outcomes. J. Appl. Psychol..

[28] Fong V.C., Iarocci G. (2020). Child and Family Outcomes Following Pandemics: A Systematic Review and Recommendations on COVID-19 Policies. J. Pediatr. Psychol..

[29] Chen C., Wittenberg E., Sullivan S.S., Lorenz R.A., Chang Y.-P. (2021). The Experiences of Family Members of Ventilated COVID-19 Patients in the Intensive Care Unit: A Qualitative Study. Am. J. Hosp. Palliat. Care.

[30] Drake R.E., Sederer L.I., Becker D.R., Bond G.R. (2021). COVID-19, Unemployment, and Behavioral Health Conditions: The Need for Supported Employment. Adm. Policy Ment. Health.

[31] Liu S., Heinzel S., Haucke M.N., Heinz A. (2021). Increased Psychological Distress, Loneliness, and Unemployment in the Spread of COVID-19 over 6 Months in Germany. Medicina.

[32] Posel D., Oyenubi A., Kollamparambil U. (2021). Job Loss and Mental Health during the COVID-19 Lockdown: Evidence from South Africa. PLoS ONE.

[33] Arnout B.A., Al-Dabbagh Z.S., Al Eid N.A., Al Eid M.A., Al-Musaibeh S.S., Al-Miqtiq M.N., Alamri A.S., Al-Zeyad G.M. (2020). The Effects of Corona Virus (COVID-19) Outbreak on the Individuals’ Mental Health and on the Decision Makers: A Comparative Epidemiological Study. Int. J. Med. Res. Health Sci..

[34] Losada-Baltar A., Jiménez-Gonzalo L., Gallego-Alberto L., Pedroso-Chaparro M.D.S., Fernandes-Pires J., Márquez-González M. (2021). “We Are Staying at Home.” Association of Self-Perceptions of Aging, Personal and Family Resources, and Loneliness With Psychological Distress during the Lock-Down Period of COVID-19. J. Gerontol. B. Psychol. Sci. Soc. Sci..

[35] López J., Pérez-Rojo G., Noriega C., Velasco C., Carretero I., López-Frutos P., Galarraga L. (2021). COVID-19 Pandemic Lockdown Responses from an Emotional Perspective: Family Function as a Differential Pattern among Older Adults. Behav. Psychol. Psicol. Conduct..

[36] Vanderhout S.M., Birken C.S., Wong P., Kelleher S., Weir S., Maguire J.L. (2020). Family Perspectives of COVID-19 Research. Res. Involv. Engagem..

[37] Gayatri M., Irawaty D.K. (2021). Family Resilience during COVID-19 Pandemic: A Literature Review. Fam. J..

[38] Rawal G., Yadav S., Kumar R. (2017). Post-Intensive Care Syndrome: An Overview. J. Transl. Intern. Med..

[39] Ghaffari Darab M., Keshavarz K., Sadeghi E., Shahmohamadi J., Kavosi Z. (2021). The Economic Burden of Coronavirus Disease 2019 (COVID-19): Evidence from Iran. BMC Health Serv. Res..

[40] Richards F., Kodjamanova P., Chen X., Li N., Atanasov P., Bennetts L., Patterson B.J., Yektashenas B., Mesa-Frias M., Tronczynski K. (2022). Economic Burden of COVID-19: A Systematic Review. Clin. Outcomes Res..

[41] Tabuñar S.M.S., Dominado T.M.P. (2021). Hospitalization Expenditure of COVID-19 Patients at the University of the Philippines-Philippine General Hospital (UP-PGH) with PhilHealth Coverage. Acta. Med. Philipp..

[42] Department of Labor and Employment National Capital Region | National Wages Productivity Commission. https://nwpc.dole.gov.ph/regionandwages/national-capital-region/.

[43] Núñez A., Sreeganga S.D., Ramaprasad A. (2021). Access to Healthcare during COVID-19. Int. J. Environ. Res. Public Health.

[44] Okereke M., Ukor N.A., Adebisi Y.A., Ogunkola I.O., Favour Iyagbaye E., Adiela Owhor G., Lucero-Prisno D.E. (2021). 3rd Impact of COVID-19 on Access to Healthcare in Low- and Middle-Income Countries: Current Evidence and Future Recommendations. Int. J. Health Plann. Manag..

[45] Singu S., Acharya A., Challagundla K., Byrareddy S.N. (2020). Impact of Social Determinants of Health on the Emerging COVID-19 Pandemic in the United States. Front. Public Health.

[46] Shadmi E., Chen Y., Dourado I., Faran-Perach I., Furler J., Hangoma P., Hanvoravongchai P., Obando C., Petrosyan V., Rao K.D. (2020). Health Equity and COVID-19: Global Perspectives. Int. J. Equity Health.

[47] Bjelland I., Krokstad S., Mykletun A., Dahl A.A., Tell G.S., Tambs K. (2008). Does a Higher Educational Level Protect against Anxiety and Depression? The HUNT Study. Soc. Sci. Med..

[48] Erickson J., El-Gabalawy R., Palitsky D., Patten S., Mackenzie C.S., Stein M.B., Sareen J. (2016). Educational attainment as a protective factor for psychiatric disorders: Findings from a nationally representative longitudinal study. Depress. Anxiety.

[49] Carlson R.H., McChesney C.S. (2014). Income Sustainability through Educational Attainment. J. Educ. Train. Stud..

[50] Wang G.-Y., Tang S.-F. (2020). Perceived Psychosocial Health and Its Sociodemographic Correlates in Times of the COVID-19 Pandemic: A Community-Based Online Study in China. Infect. Dis. Poverty.

