# Supplementary material for: Psychosocial Distress among Family Members of COVID-19 Patients Admitted to Hospital and Isolation Facilities in the Philippines: A Prospective Cohort Study
Source: J Clin Med. 2022 Sep 5;11(17):5236. doi: 10.3390/jcm11175236 (PMC9457412; doi:10.3390/jcm11175236)
Supplement: Supplementary file 1 [file jcm-11-05236-s001.zip › jcm-1831566-supplementary.pdf]

Supplemental Table S1. Instrument scores of patients' family members at 2 and 8 weeks after discharge of their relatives with COVID-19 from the study sites (N = 74).

**Instruments**	**Scores**				***P value***
	**2 weeks**		**8 weeks**		
	**Mean**	**SD**	**Mean**	**SD**	
HADS-P Anxiety	7.24	4.60	5.04	3.83	< 0.001
HADS-P Depression	4.28	3.79	3.31	3.05	0.007
Family APGAR	9.34	1.17	9.24	1.73	0.604
SCREEM RES	26.27	4.47	25.99	4.71	0.547
